# The gibberellin GID1-DELLA signalling module exists in evolutionarily ancient conifers

**DOI:** 10.1038/s41598-017-11859-w

**Published:** 2017-11-30

**Authors:** Ran Du, Shihui Niu, Yang Liu, Xinrui Sun, Ilga Porth, Yousry A. El-Kassaby, Wei Li

**Affiliations:** 1 Beijing Advanced Innovation Center for Tree Breeding by Molecular Design, National Engineering Laboratory for Forest Tree Breeding, College of biological sciences and technology, Beijing Forestry University, Beijing, 100083 P.R. China; 2 Science and Technology Development Center, State Forestry Administration, Beijing, 100714 P.R. China; 3 Département des sciences du bois et de la forêt, Faculté de foresterie, de géographie et de géomatique, Université Laval, 1030 Avenue de la Médecine, Québec, Québec, G1V 0A6 Canada; 4 Department of Forest Sciences, Faculty of Forestry, The University of British Columbia, 2424 Main Mall, Vancouver, British Columbia V6T 1Z4 Canada

## Abstract

Gibberellins (GAs) participate in controlling various aspects of basic plant growth responses. With the exception of bryophytes, GA signalling in land plants, such as lycophytes, ferns and angiosperms, is mediated via GIBBERELLIN-INSENSITIVE DWARF1 (GID1) and DELLA proteins. To explore whether this GID1-DELLA mechanism is present in pines, we cloned an orthologue (*PtGID1*) of *Arabidopsis AtGID1a* and two putative DELLA proteins (PtDPL; PtRGA) from *Pinus tabuliformis*, a widespread indigenous conifer species in China, and studied their recombinant proteins. PtGID1 shares with AtGID1a the conserved HSL motifs for GA binding and an N-terminal feature that are essential for interaction with DELLA proteins. Indeed, *A*. *thaliana 35S:PtGID1* overexpressors showed a strong GA-hypersensitive phenotype compared to the wild type. Interactions between PtGID1 and PtDELLAs, but also interactions between the conifer-angiosperm counterparts (i.e. between AtGID1 and PtDELLAs and between PtGID1 and AtDELLA), were detected *in vivo*. This demonstrates that pine has functional GID1-DELLA components. The Δ17-domains within PtDPL and PtRGA were identified as potential interaction sites within PtDELLAs. Our results show that PtGID1 has the ability to interact with DELLA and functions as a GA receptor. Thus, a GA-GID1-DELLA signalling module also operates in evolutionarily ancient conifers.

## Introduction

Gibberellins (GAs) are a class of phytohormones that function in a wide range of basic plant growth responses. These GA-mediated responses include seed germination, stem elongation, shade avoidance (in competitive interactions), leaf expansion, pollen maturation, and induction of flowering^1–4^, but GAs can also induce temporary growth arrest under adverse environmental conditions^5^. Regarding our understanding of the GA signalling pathways *in planta*, progress has been made in the past few years primarily in rice^6^ and *Arabidopsis thaliana* ^7^.

Angiosperms use the GA-GID1 (GIBBERELLIN-INSENSITIVE DWARF1)-DELLA pathway, which involves the nuclear GA receptor GID1^8^, the repressor DELLA protein^9,10^, and the F-box protein GID2/SLY1, which degrades the repressor DELLA protein to trigger GA-mediated downstream responses^5,11,12^. Although it has not yet been thoroughly studied, GA signalling in conifers should also follow the GID1-DELLA pathway; this molecule is present in early vascular plants such as the lycophyte *Selaginella moellendorffii* ^13^, but not in non-vascular bryophytes^14^. Hence, GID1-mediated GA signalling likely appeared after the divergence of vascular plants from mosses, which took place ~430 million years ago^15^.

*Arabidopsis thaliana* possesses at least 10 similar DNA sequences for GID1, of which only three (*AtGID1a*, *AtGID1b*, and *AtGID1c*) encode proteins that function as GA receptors^16^; conversely, there is only one GID1 gene in rice (*OsGID1*). *At*GID1b is unique in that it can also interact with DELLAs in the absence of GA^17^, indicating that there are GA-independent pathways for this interaction. However, *GID1* overexpression leads to a GA overdose phenotype, where plants are tall with long, light green leaves, fewer tillers, and dramatically reduced fertilities^8^. Leaf expansion, and stem and root elongation are reduced in *gid1*-knockout mutants, which is consistent with the GA-deficient phenotype^18^.

At the molecular level, GID1 displays an alpha/beta-hydrolase fold characteristic of hormone-sensitive lipases (HSLs), and its GA-binding pocket also corresponds to the substrate-binding site of HSLs; however, the N-terminal lid is specific to GID1 proteins^19^. The primary function of this movable N-terminal lid is to stabilise GA within the GID1 binding site^20^. The N-terminal lid also participates in GA-dependent interactions with DELLA proteins^20^, leading to the formation of GA-GID1-DELLA protein complexes^5^.

Rice contains only a single DELLA protein, SLR1, whose causative recessive mutation is responsible for the ‘slender rice’ constitutive GA response phenotype^21^. In contrast, *A*. *thaliana* has five DELLA family members with partly overlapping functions: GA-INSENSITIVE (GAI), REPRESSOR OF *ga1-3* (RGA), and three RGA-like proteins (RGL1, RGL2, and RGL3)^22^. A 17-amino-acid deletion(Δ17), DELLAVLGYKVRSSEMA, within the DELLA region turns proteins into constitutive repressors of GA signalling, wherein DELLA fails to interact with GID1 in the presence of GA, conferring a GA-insensitive dwarf phenotype^9,23^.

The typical molecular features of the GA-signalling regulatory DELLA proteins include the conserved N-terminal DELLA and TVHYNP motifs, which are required for GA binding and for inactivation of DELLA proteins^6^, and the conserved C-terminal GRAS domain, which interacts with GID1^19,22^.

Recent findings suggest that DELLA controls the GA signalling pathway through antagonistic regulation of the GA-positive regulator SCARECROW-LIKE 3 (SCL3) promoter sequence via co-regulatory intermediary proteins^24^. DELLAs also have a more general function in adapting plant growth to environmental conditions. For example, salt-activated signalling pathways (via abscisic acid and ethylene) enhance the growth-repressing effects of DELLAs^25^.

The objective of the present study was to investigate whether the GA-GID1-DELLA module is also present in gymnosperms. Our study species was *Pinus tabuliformis*, an economically and ecologically important indigenous hard pine of Northern China^26^. Characteristic of conifers, *P*. *tabuliformis* has a long growing cycle, a huge genome (Mean DNA content = 25.7 ± 0.13 Gb)^27^, and a large evolutionary separation from angiosperms^28^. Our primary long-term research focus is the characterisation of the regulatory program underlying pine cone development. We have previously studied the patterns of expression for GA metabolism genes and found that GA plays different roles in the early and late stages of cone development^26^. We have also isolated and identified the MADS-box genes and their potential regulators related to reproductive development on a genome-wide basis^28^. Furthermore, we have systematically isolated the pine homologues of the functional genes from the miRNA pathway involved in cone development^29^. In these previous studies, we discovered that the GA biosynthesis pathway has diverged remarkably between conifers and angiosperms^26^. To determine which components of the GA signalling pathway are conserved between conifers and angiosperms, we assessed the potential involvement of a conifer GA-GID1-DELLA module for cone development in *Pinus tabuliformis*.

## Results

### The *P*. *tabuliformis* orthologue of *AtGID1a* and DELLA proteins

**Figure 1:**
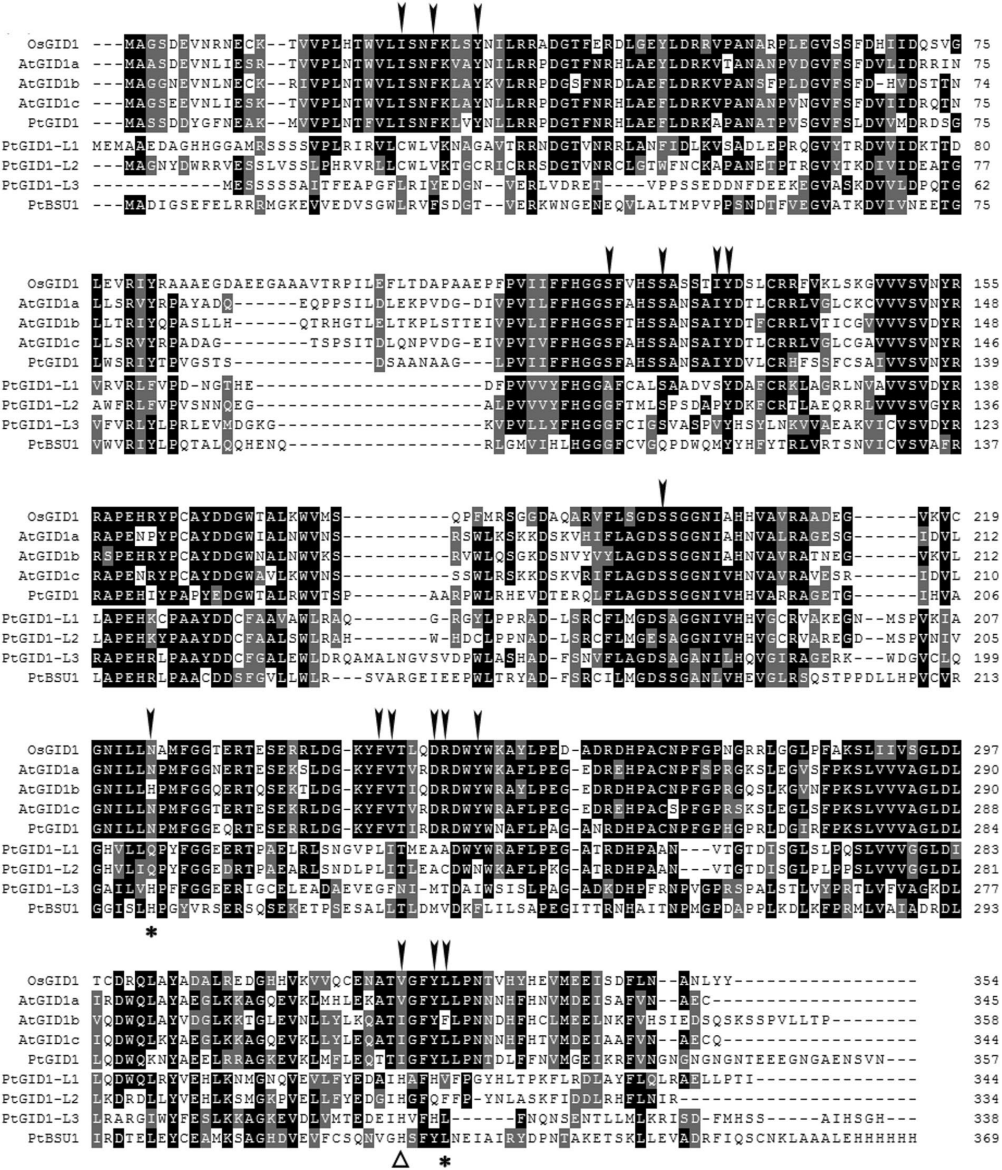
Multiple sequence alignment of protein sequences encoded by *PtGID1-like* genes and *A*. *thaliana* and *O*. *sativa GID1* genes. Black and gray boxes indicate identical or similar residues. The 17 arrows at the top indicate the residues essential for the gibberellic acid binding activity of GID1 in angiosperms. The two asterisks at the bottom indicate sites that are not identical for *PtGID1-like* genes within the conserved sequence region found in angiosperms. The triangle indicates similar residues in AtGID1b, AtGID1c, and PtGID1, and in OsGID1 and AtGID1a. As in rice and *Arabidopsis*, three conserved amino acids (S, D, and H) shape the catalytic triad in the HSL family (Nakajima *et al*.^16^; Ueguchi-Tanaka *et al*.^8^). Two of them (S and D) are conserved in *Pt*GID1, while the third (H) is replaced by V or I. There are 13 functional domains (TWVLIS, LDR, FFHGGSF, HS, IYD, YRR, DGW, GDSSGGNI, GNI, MF, LDGKYF, WYW, and GFY) in GID1 (Hirano *et al*.^14^). Within TWVLIS, W is replaced by F, while all other domains are conserved. We confirmed the presence of all amino acid residues essential for GA binding encoded within the cloned *PtGID1* gene.

Based on *P*. *tabuliformis* transcriptome data (SRA 056887) and a large collection of high-quality ESTs that was obtained, assembled *de novo* and characterised^30^, we screened and cloned five *GID1* homologue sequences: *PtGID1*, *PtGIDI-L1*, *PtGIDI-L2*, *PtGIDI-L3*, and *PtBSU1*. Phylogenetic reconstruction revealed that only *PtGID1* was the true orthologue of *AtGID1a* and *OsGID1* (Figure S1). The *PtGID1* gene encodes a 357-amino-acid-long polypeptide, and has the same gene structure as the *A*. *thaliana* and rice genes, containing one intron and two exons. *PtGID1* shares homology with the consensus sequence of the HSL family, including the conserved HSL motifs HGG and GXSXG. Multiple sequence alignment revealed that 16 of the 17 essential sites in PtGID1 are identical to those found within OsGID1, AtGID1a, and AtGID1c, while valine (V) at position C326 (following *Os*GID1’s amino acid sequence as the reference sequence in the alignment) is replaced by isoleucine (I) (Fig. 1, Δ). Nevertheless, both amino acids have similar hydrophobic properties. The results of comparative sequence analysis with functional *GID1* from angiosperms suggested that the isolated *PtGID1* gene might indeed encode the functional GA receptor for GA-dependent signalling pathways in pine. Homology modelling for PtGID1 further showed that the PtGID1 core domain is similar to that of OsGID1 based on crystal structure-derived information for the rice orthologue (Figure S2). The GA binding site of PtGID1 has one additional α-helix compared to OsGID1. However, this N-terminal extension (N-Ex) is the same as in AtGID1a^20^, demonstrating that the structure of the GA-binding sites of PtGID1, OsGID1, and the AtGID1s are highly conserved during evolution. These findings indicate that the *PtGID1* gene might encode fully functional GA signal transduction capability.

**Figure 2:**
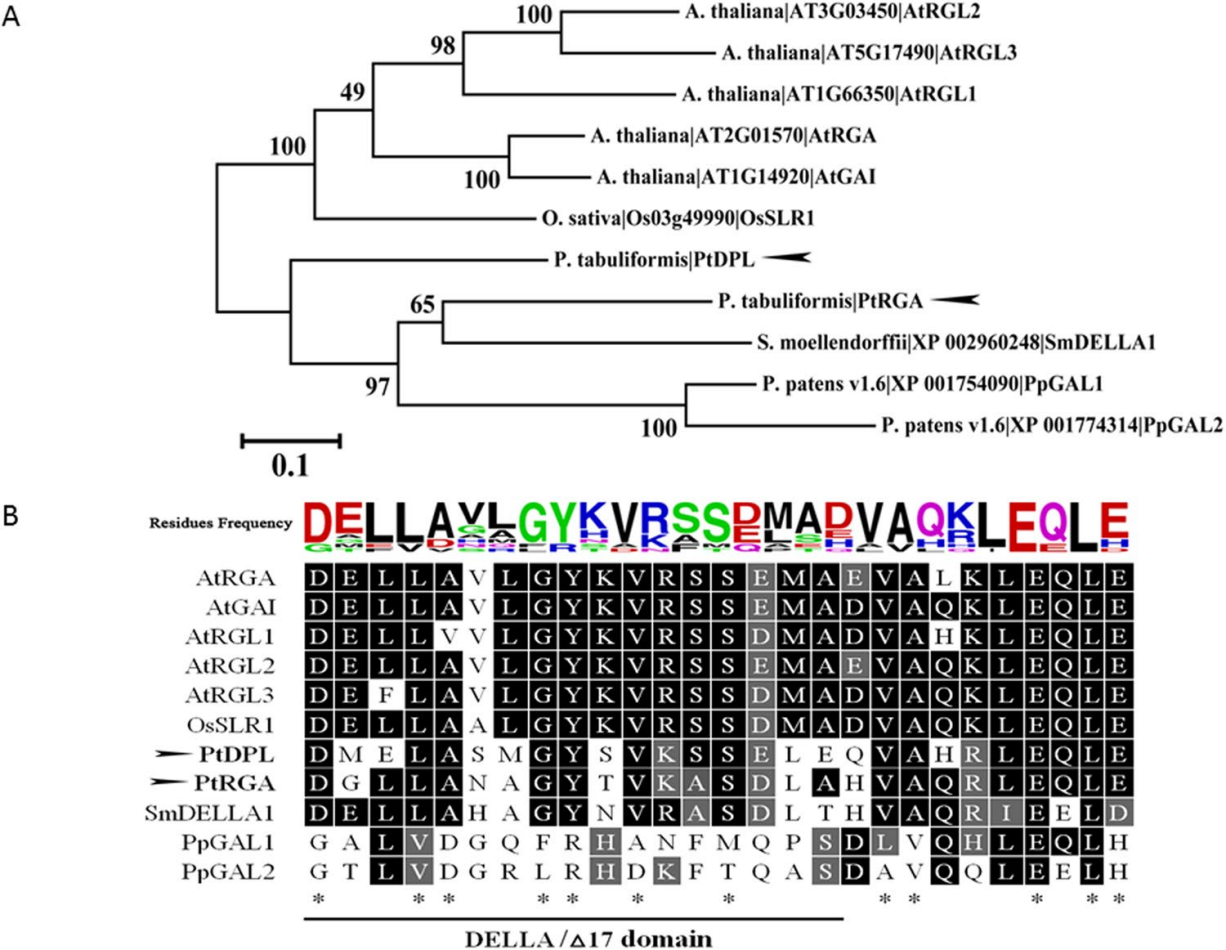
Comparative sequence analysis for 11 DELLA proteins or homologues in angiosperms with or without vascular tissue (rice, *Arabidopsis*, *Selaginella*, and moss) and conifers (pine). (**A**) Phylogenetic analysis of DELLA proteins or homologues. The maximum likelihood tree is based on the complete amino acid sequences of DELLA proteins from *P*. *tabuliformis*, *A*. *thaliana*, *P*. *patens*, *S*. *moellendorffii*, and *O*. *sativa*. The species, and gene names and IDs are displayed on the right side of each branch. Bootstrap values were obtained by running 1,000 bootstrap replicates. The horizontal branch lengths are proportional to the estimated number of amino acid substitutions per residue. The arrows indicate *P*. *tabuliformis* genes isolated in the present study. (**B**) DELLA domain sequence alignment for DELLA homologues in *A*. *thaliana*, *O*. *sativa*, *P*. *patens*, *S*. *moellendorffii*, and *P*. *tabuliformis*. Black and gray boxes indicate identical or similar residues, respectively. Asterisks at the bottom represent identical residues. The DELLA Δ17 domain range is depicted by a black line at the bottom. The sizes of the letters above the sequence alignment represent the residue frequency at each site for the 11 studied gene sequences. The arrows indicate the two *P*. *tabuliformis* genes isolated for this study.

We identified two candidates from high-quality *P*. *tabuliformis* ESTs^30^ and designated the encoded proteins as PtDPL and PtRGA based on their specific DELLA and GRAS domains, which were indicative of DELLA proteins. Phylogenetic reconstruction revealed a greater evolutionary distance between PtRGA and angiosperms than between PtRGA and the spike moss *Selaginella* (Fig. 2A). However, it has already been shown that the spike moss protein (SmDELLA1) is capable of interacting with its GID2 protein as a component in the GID-dependent GA signalling pathway. Functionally characterised angiosperm DELLA proteins have a conserved domain that is 27 amino acids in length, starting with the aspartic acid, glutamic acid, leucine, leucine, and alanine (“DELLA”) sequence that lends the protein family its specific name (Fig. 2B). This sequence can then interact with the GID1 protein N-Ex sequence. In the absence of the Δ17-domain (Fig. 2B), GID1 is unable to recognise DELLA, leading to the interruption of GA signal transmission^31^. In *P*. *tabuliformis*, seven amino acid sites within the DELLA Δ17-domains of both PtDPL and PtRGA are identical to those within the respective angiosperm proteins (Fig. 2B). Thus, these residues may play a crucial role in GID1 recognition and its interactions, as they have been evolutionarily conserved.

### *PtGID1* expression is regulated by GA

**Figure 3:**
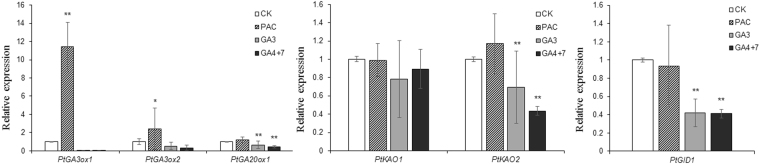
*PtGID1* expression is decreased in *P*. *tabuliformis* after GA treatment. Relative expression levels of the GA biosynthesis genes *PtGA3ox1*, *PtGA3ox2*, *PtGA20ox1*, *PtKAO1*, and *PtKAO2* and the GA receptor *PtGID1*, determined by real-time RT-PCR in 2-month-old pine after treatment with 50 μM GA_3_, 50 μM GA_4+7_, or 50 μM PAC. The expression levels were normalised to 18S rRNA. The data are shown as the means ± SD of biological triplicates.

The GA metabolism genes in *P*. *tabuliformis* were isolated and identified in a previous study, in which *PtGA20ox* and *PtGA3ox* were shown to catalyse the penultimate and final steps, respectively, and *ent-kaurenoic acid oxidase* (*PtKAO*) was shown to be expressed upstream^26^. We measured the expression levels of *PtGA3ox*, *PtGA20ox*, *PtKAO*, and *PtGID1* in *P*. *tabuliformis* after GA_3_, GA_4+7_, or treatment with the GA biosynthesis inhibitor paclobutrazol (PAC) (Fig. 3). PAC treatment resulted high expression levels of *PtGA3ox*. *PtKAO2* mRNA levels were significantly lower than in non-treated pine needles when exogenous GA was present. This indicated that GA and PAC treatment affected the GA feedback mechanism. *PtGID1* had dramatically lower expression levels following the application of either GA_3_ or GA_4+7_ than non-treated pine needles, and higher expression levels after PAC treatment. This showed that *PtGID1* had functional GA signal transduction capability.

### *PtGID1* confers a GA-sensitive phenotype

**Figure 4:**
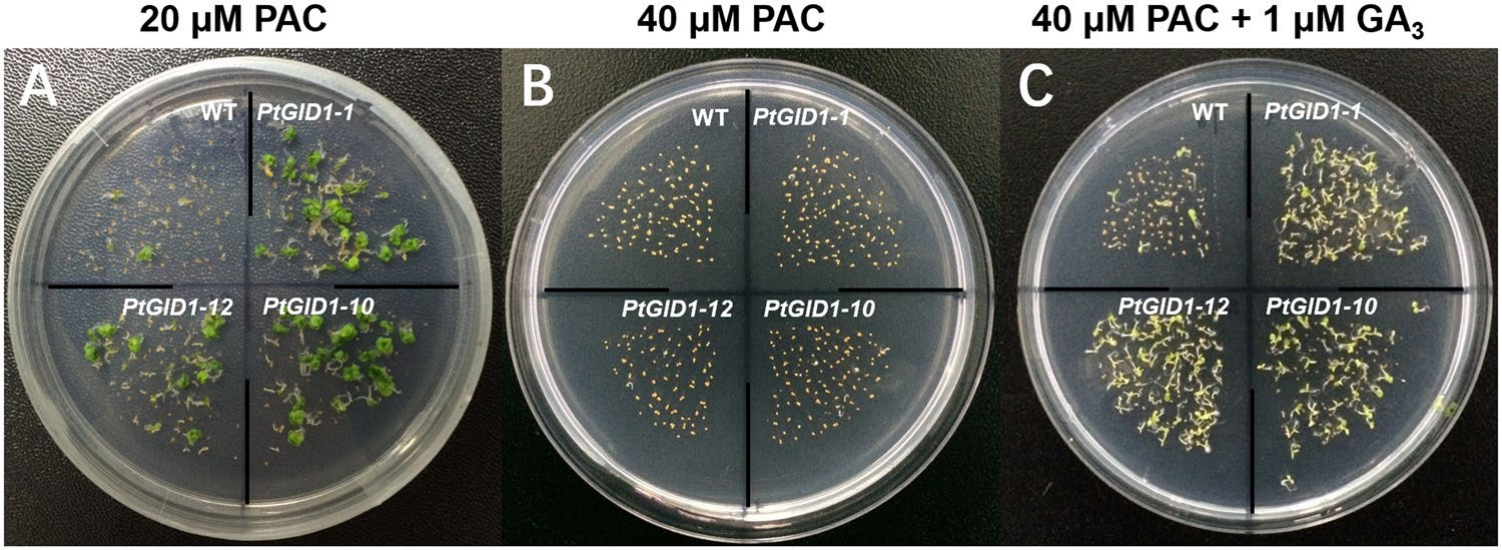
*PtGID1* overexpression rescues *Arabidopsis* plants grown on medium containing the GA biosynthesis inhibitor PAC. Wild-type (WT) plants and three lines of *PtGID1* plants (*PtGID1-1*, *PtGID1-10*, and *PtGID1-12*) were sown on MS-agar with (**A**) 20 μM PAC, (**B**) 40 μM PAC, or (**C**) 40 μM PAC and 1 μM GA_3_, and incubated at 22 °C. Scale bar, 1 cm.

To establish that *PtGID1* genes encode a GA receptor function *in vivo*, we generated *A*. *thaliana* transformants overexpressing the *PtGID1* gene under the control of the constitutive 35S promoter. Seeds from wild-type *A*. *thaliana*, and from three lines of *PtGID1*-overexpressing *A*. *thaliana* (*PtGID1-1*, *PtGID1-10*, and *PtGID1-12*), were sown with medium containing low PAC concentrations (20 μM). Wild-type growth was suppressed under these conditions, but most seeds from the three *PtGID1*-overexpressor lines germinated (Fig. 4A). Because the three *PtGID1*-overexpressor lines had a higher rate of germination than the wild type, *PtGID1* overexpressors presumably have a greater capability to perceive endogenous GA than the wild type. After increasing PAC concentration to 40 μM, growth was repressed in all cases (control case, Fig. 4B). Wild-type growth was repressed with the application of exogenous GA_3_, but *PtGID1* overexpressors germinated normally (Fig. 4C)_._ This indicated that *PtGID1* overexpressors are better able to detect GA and are more efficient in GA signal transduction than the wild type.

**Figure 5:**
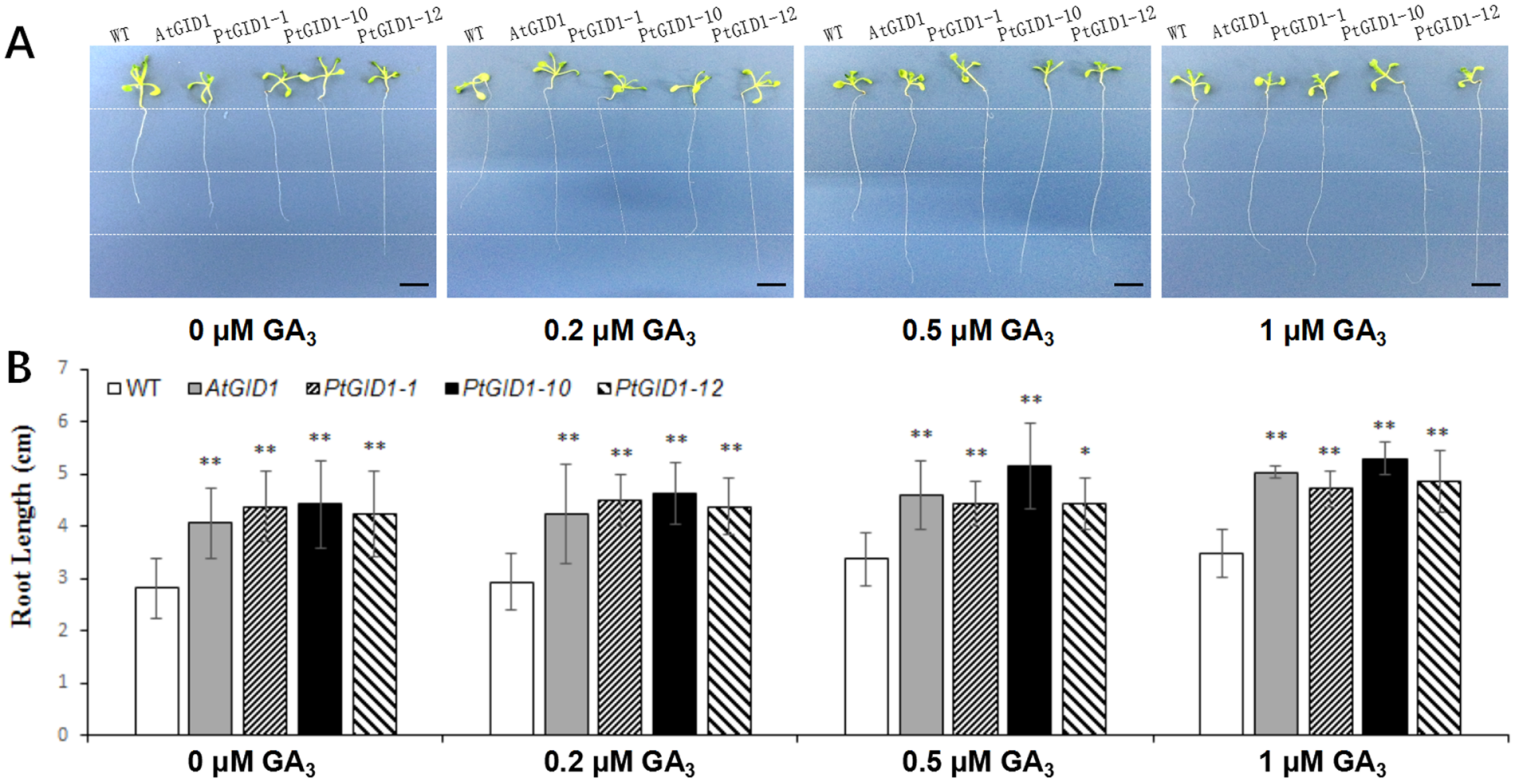
*PtGID1* overexpression in *Arabidopsis* plants promotes root elongation under GA application. (**A**) Representative 6-day-old seedling primary roots of WT, *PtGID1-1*, *PtGID1-10*, and *PtGID1-12* seedlings grown on MS-agar with GA_3_-free, 0.2, 0.5, or 1 μM GA_3_. Scale bar, 1 cm (n = 36). (**B**) Mean lengths in cm (mean ± SD, n = 36) of WT, *PtGID1-1*, *PtGID1-10*, and *PtGID1-12* seedlings grown on MS-agar with GA_3_-free, 0.2, 0.5, or 1 μM GA_3_ are presented.

The opposing functions of DELLA proteins and GA in regulating root growth have been reported^32^. However, it remains unclear whether *PtGID1* (which degrades DELLA proteins) exhibits sensitive GA-binding activity and influences root growth. To investigate this question, we created *AtGID1a* overexpressors. Figure 5A shows root growth of the wild type, *AtGID1a* overexpressor, and three independent lines of the *PtGID1*-overexpressing plants (*PtGID1-1*, *PtGID1-10*, and *PtGID1-12*). All plants were grown on GA_3_-free medium or on medium supplemented with 0.2, 0.5, or 1 μM GA_3_. We observed greater root elongation in all lines with increasing exogenous GA_3_ concentrations, but root elongation in the wild type was significantly and consistently lower than in the *AtGID1* and *PtGID1* overexpressors (Fig. 5B). This indicated a GA-hypersensitive phenotype in the transformants and confirmed that *PtGID1* is a GA receptor in *P*. *tabuliformis*.

**Figure 6:**
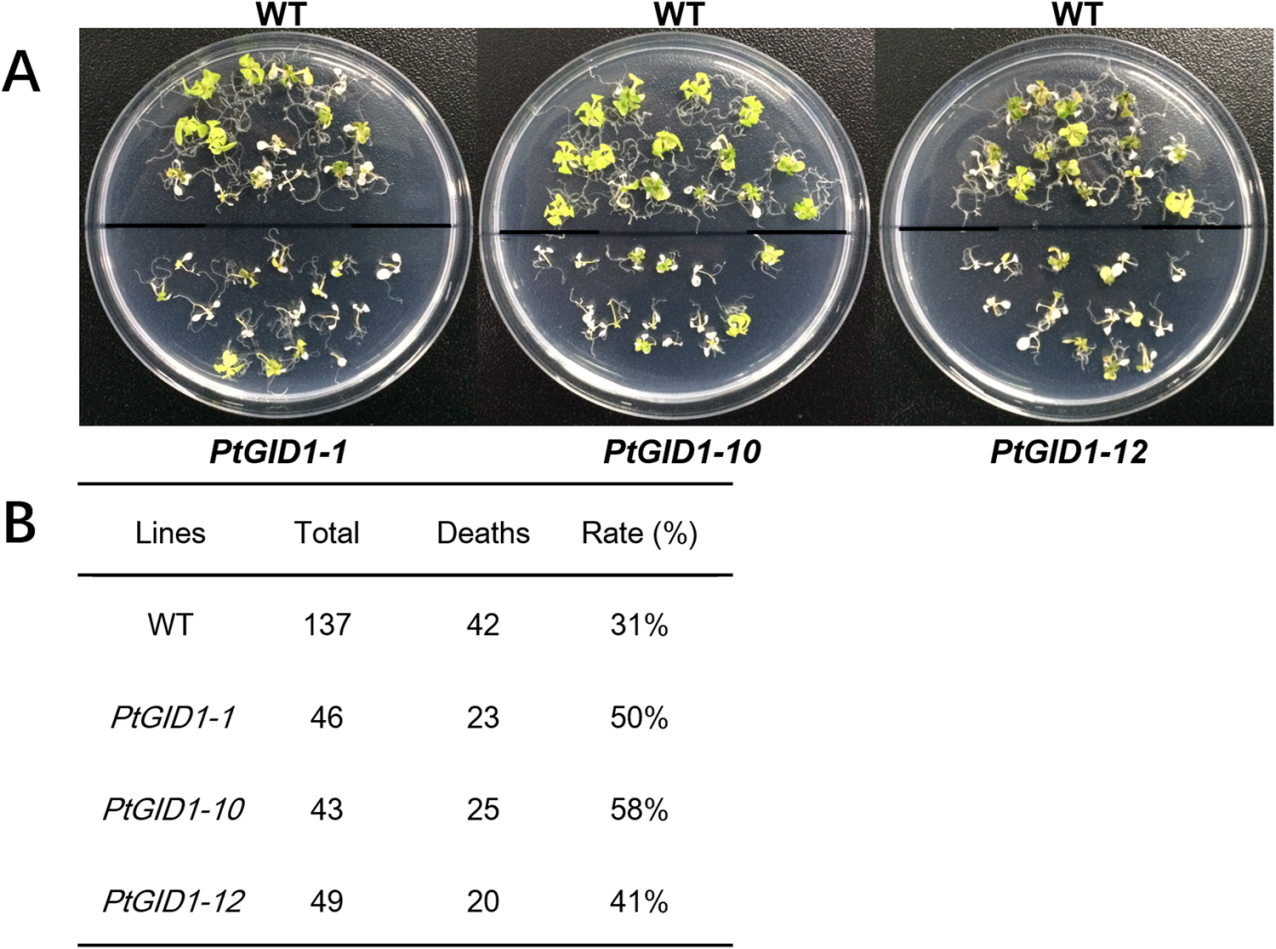
Non-survival rates of *A*. *thaliana PtGID1* overexpressors at toxic salt concentrations. (**A**) Representation of survival among WT, *PtGID1-1*, *PtGID1-10*, and *PtGID1-12* transformants grown on high-salt (150 mM) medium. Photographs were taken 20 days after plants had been transferred to high-salt medium. Live plants are green; dead plants are white. (**B**) Numbers of WT, *PtGID-1*, *PtGID-10*, and *PtGID-12* plants that failed to grow on high-salt medium (expressed as total number of dead plants and rate of non-survival in %).

In *A*. *thaliana*, it has been suggested that DELLA proteins suppress plant growth to enhance survival in saline environments^5,9,33^. We therefore investigated the role of the GA receptor in growth and salt tolerance by promoting the degradation of DELLAs (Fig. 6A). We found that all three *PtGID1* overexpressor lines were less viable than the wild type under conditions of high salt stress (Fig. 6B). Thus, we surmised that *PtGID1* was involved in the GA-GID1-DELLA module, and that the PtGID1-GA protein complex stimulates growth by promoting the degradation of DELLAs.

**Figure 7:**
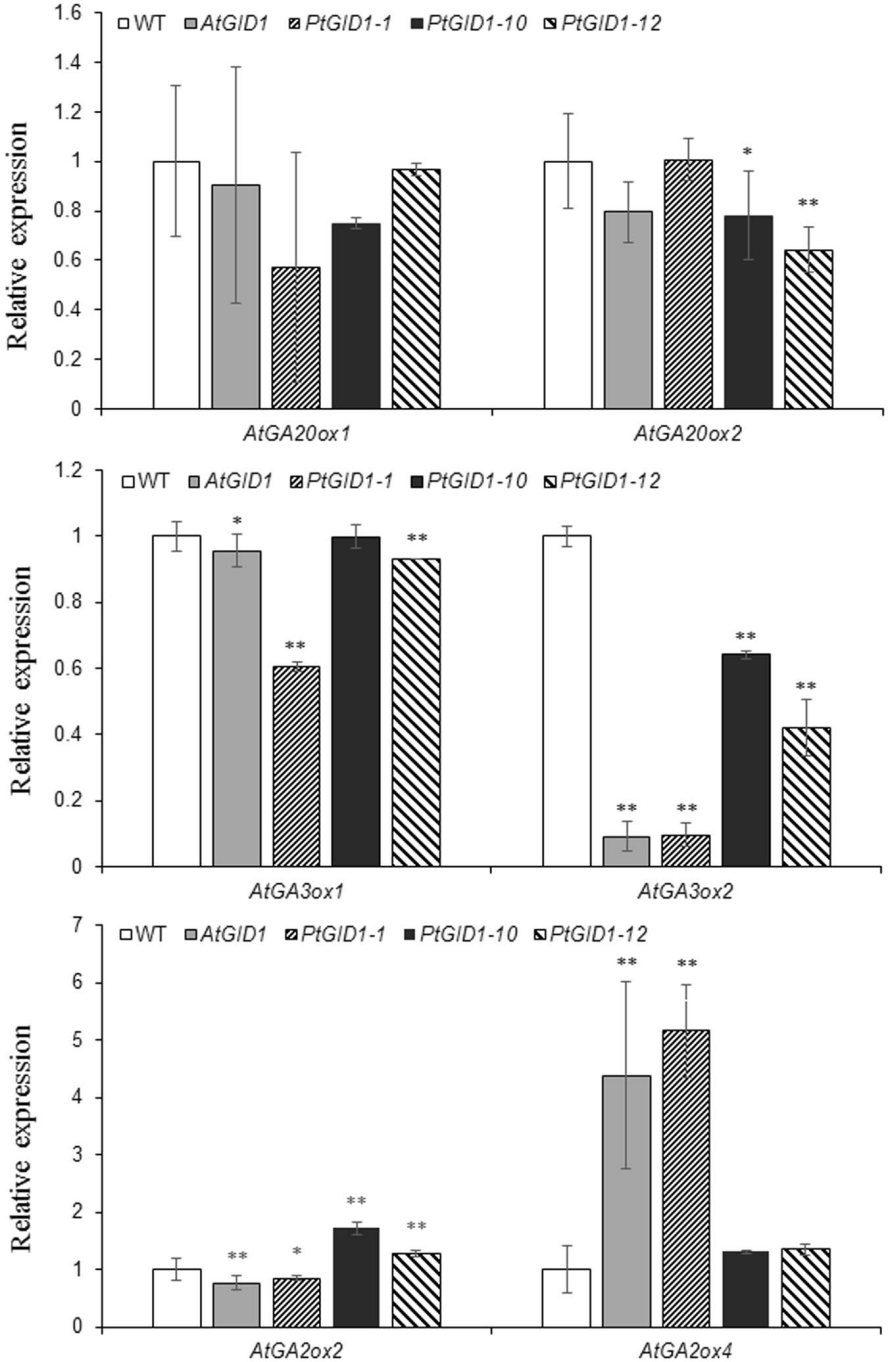
*PtGID1* overexpressors upregulate *GA 2-oxidase* transcript levels. Relative expression levels of GA biosynthesis *AtGA3ox1*, *AtGA3ox2*, *AtGA20ox1*, and *AtGA20ox2* transcripts and GA deactivation of *AtGA2ox2*, *AtGA2ox4* gene transcripts levels (determined by real-time RT-PCR) in 8-d-old seedlings of WT, *AtGID1*, *PtGID1-1*, *PtGID1-10*, and *PtGID1-12* plants. The expression levels were normalised to 18S rRNA. The data are shown as the means ± SD of biological triplicates.

In the GA metabolism pathway of *Arabidopsis*, GA 20-oxidase (AtGA20ox) and GA 3-oxidase (AtGA3ox) catalyse successive steps in the synthesis of bioactive GAs^34^. However, AtGA2ox also deactivates bioactive GAs^35^. We assessed the levels of mRNA expression for *AtGA20ox*, *AtGA3ox*, and *AtGA2ox* in wild-type plants, as well as in *GID1*-transgenic plants (Fig. 7). We found that both *AtGID1* overexpressors and *PtGID1* overexpressors contained lower levels of *AtGA3ox1*, *AtGA3ox2*, *AtGA20ox1*, and *AtGA20ox2* transcripts than wild-type plants. In contrast, *GID1* overexpressors exhibited highly increased levels of *AtGA2ox2* and *AtGA2ox4* expression. Thus, the overexpression of *PtGID1*, like *AtGID1*, increased the sensitivity of *Arabidopsis* to GA.

### Angiosperms and evolutionarily distant conifers share similar GID-DELLA modules

**Figure 8:**
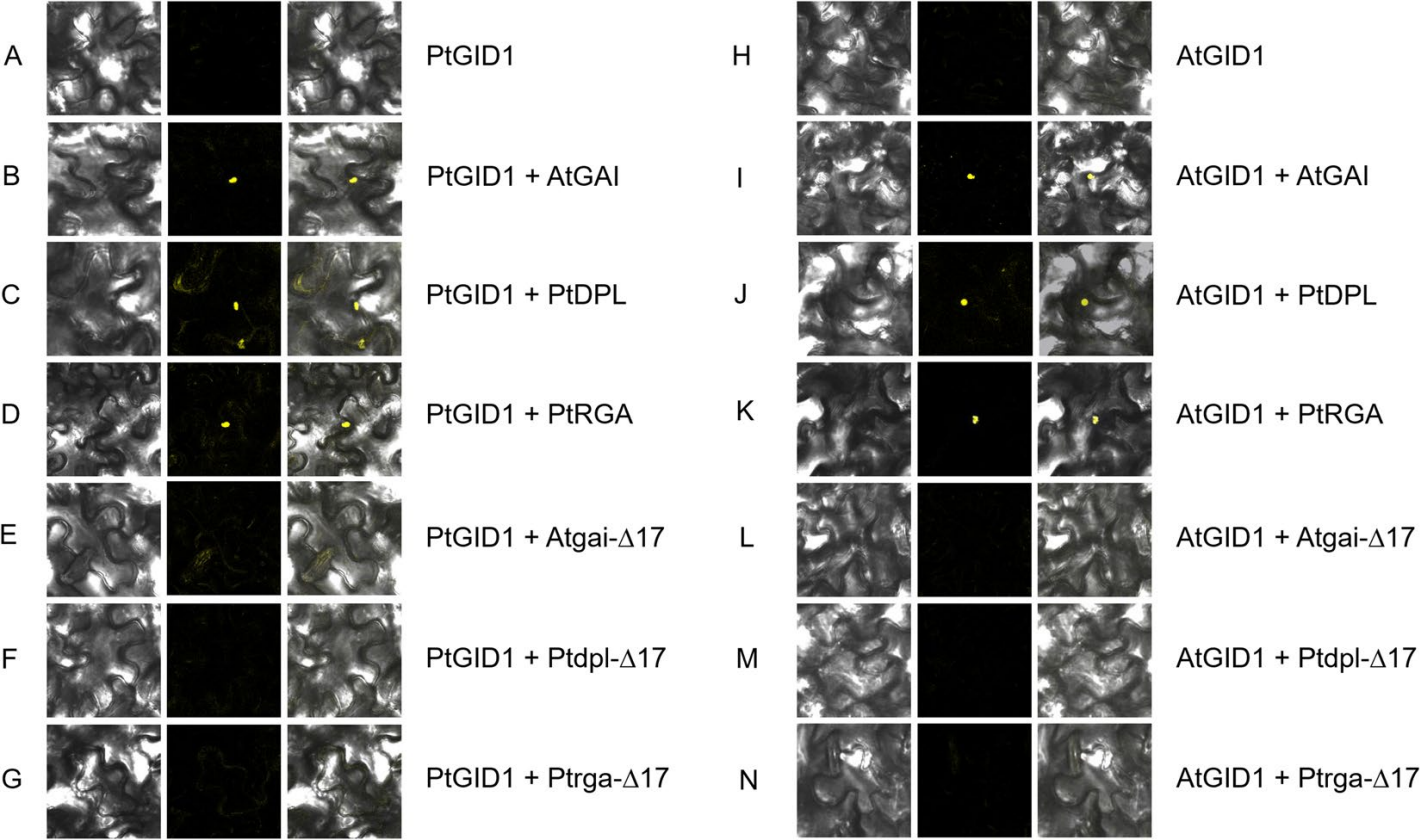
BiFC analysis of the GID1-DELLA interaction in nuclei of transfected *A*. *thaliana*. AtGID1 and PtGID1 were expressed and interactions tested with wild-type and mutant *A*. *thaliana* and *P*. *tabuliformis* DELLA proteins (AtGAI, Atgai; PtDPL, Ptdpl; PtRGA; Ptrga), respectively. Bright-field image, YFP fluorescence image, and the merged image are each displayed for expression of PtGID1 alone (**A**); co-expression with PtDPL (**B**), AtGAI (**C**), PtRGA (**D**), Δ17-domain mutant Ptdpl (**E**), Δ17-domain mutant Atgai (**F**), and Δ17-domain mutant Ptrga (**G**); Bright-field image, YFP fluorescence image, and the merged image are each displayed for expression of AtGID1 alone (**H**); co-expression with PtDPL (**I**), AtGAI (**J**), PtRGA (**K**), Δ17-domain mutant Ptdpl (**L**), Δ17-domain mutant Atgai (**M**), and Δ17-domain mutant Ptrga (**N**).

While previous studies have thoroughly elucidated the interaction between AtGID1 and DELLA proteins, to date no such studies have focused on conifers. We performed yeast two-hybrid assays, and observed that PtDPL and PtRGA had self-activating functions, which is consistent with previous reports^36^. We also performed bimolecular fluorescence complementation (BiFC) experiments to test whether PtGID1 and DELLA proteins interact *in vivo*. Each was fused to the reporter yellow fluorescence protein (YFP) which lacked either its C-terminal or its N-terminal end; thus, the YFP signal could be detected only upon interaction between PtGID1 and DELLA (Fig. 8). Fluorescence signals indicating interactions between PtGID1/AtGID1 and PtDPL, PtRGA, and AtGAI were observed in the nucleus, indicating that PtGID1 could interact with PtDPL and PtRGA (Fig. 8C,D), and that AtGID1 interacted with AtGAI (Fig. 8I). Moreover, AtGID1 could also interact with PtDPL and PtRGA (Fig. 8J,K), and PtGID1 could interact with AtGAI (Fig. 8B), suggesting that conifers and angiosperms have similar GID1-DELLA interaction patterns. To gain further insight into the sites of interaction for DELLA proteins in *P*. *tabuliformis*, we removed the identified Δ17-domains from *PtDPL*, *PtRGA*, and *AtGAI* to generate the mutated *Ptdpl*, *Ptrga*, *Atgai* sequences, respectively. We cloned these mutated sequences into expression vectors using overlap-extension PCR technology for site-directed mutagenesis. We observed no fluorescence signals corresponding to interactions between PtGID1/AtGID1 and Ptdpl, Ptrga, and Atgai (Fig. 8E,F,G,L,M,N). This supported our hypothesis that the Δ17-domain contains the site necessary for interaction. In particular, the seven amino acid residues within this Δ17 domain that are conserved between *P*. *tabuliformis* and angiosperms may be the core sites for GID1-DELLA interaction in conifers.

## Discussion

We cloned five sequences from *P*. *tabuliformis* that had high sequence homology to GID1 proteins, and confirmed that only *PtGID1* is orthologous to the *A*. *thaliana* and rice *GID1* genes. *PtGID1* shares the conserved motifs HGG and GXSXG with the HSL protein family, as well as the essential sites for binding GA and interacting with DELLA proteins. The 3D protein structure of PtGID1 is similar to that of OsGID1, while PtGID1 has an additional α-helix compared to OsGID1 and an N-ex sequence similar to AtGID1a. Thus, during the course of evolution, ancient GID1-like receptors from lycophytes and mosses have developed a pocket into which the GA molecule fits^37^. An additional innovation in conifers and angiosperms is the amino acid ‘lid’ in GID1 that holds the GA molecule in place.

Our data show that the conifer *GID1* gene is derived from genes in the HSL family, as their essential sites for protein structure and function are highly conserved in the conifer GID1 protein. This led us to hypothesise that *PtGID1* has the ability to interact with DELLA, and that it may also function as a GA receptor similar to GIDs in angiosperms. Moreover, when *P*. *tabuliformis* pine needles were treated with GA, the level of *PtGID1* expression decreased dramatically compared to the non-treated control. In other words, a common GA-GID1-DELLA signalling module may also operate in conifers.

As part of this potential GA-GID1-DELLA signalling module, we also identified two DELLA proteins from *P*. *tabuliformis*, which we termed PtDPL and PtRGA. These two DELLA proteins have a variant of the DELLA domain when compared to DELLA proteins from other higher plants/angiosperms, which is capable of interacting with the respective GID1 N-Ex. DELLA proteins are of great importance in the evolution of the GA signalling pathway: they have played a role in interacting with GID1 and regulating downstream signal transduction since the divergence from lycophytes. Seven amino acid residues within the Δ17 domain may be the core sites for GID1-DELLA interaction in *P*. *tabuliformis*. As these amino acid residues are also identical in angiosperms, they may play a crucial role in the interaction with GID1.

We also identified a GA-hypersensitive phenotype in *PtGID1* overexpressors. This indicated an increase in the ratio of inactive GID1-DELLA complex to active DELLA repressor^38^, and suggested that these mutants had an increased ability to stimulate the GA signalling pathway. In support of this theory, the *PtGID1*-overexpressor *A*. *thaliana* mutant exhibited greater root elongation compared to the wild type. This effect is consistent with a GA overdose phenotype, and is comparable to the tall *OsGID1* and *AtGID1* overexpressor phenotypes^8,16^ and to the enhanced stem elongation phenotype induced by overexpressing *PttGID* in aspen^39^. These *PtGID1* overexpressors had a reduced ability to endure salt toxicity compared to the wild type, indicating reduced salt tolerance. This supported the notion that DELLA proteins were degraded by overdosing GA-GID1 in *PtGID1* overexpressors. This finding supports the notion that DELLA proteins help to enhance survival in saline environments by repressing plant growth^25^.

The expression levels of the GA biosynthesis genes *AtGA3ox* and *AtGA20ox* were downregulated and GA deactivation of *AtGA2ox* genes was significantly increased in *PtGID1* overexpressors compared with the wild type. These effects were the same as those observed in *AtGID1* overexpressors. Thus, the changes in the expression of GA metabolism genes in *PtGID1*-transgenetic *Arabidopsis*, and the *PtGID1* gene in *P*. *tabuliformis*, showed that *PtGID1* is a GA receptor with biological function.

To demonstrate the existence of functional GID1-DELLA interaction in *P*. *tabuliformis*, we investigated several GA signalling models (Fig. 8). Using BiFC assays, we detected interactions between AtGID1 and the AtDELLA proteins, as well as between PtGID1and the PtDELLA proteins. There were no interactions in the species with Δ17-domain-mutated DELLA proteins; however, there were interactions between GID1 and DELLAs from angiosperm and conifer species. This led us to conclude that (1) conifer *P*. *tabuliformis* has functional GID1-DELLA components; (2) conifers and angiosperms have the same patterns of GID1-DELLA interaction; and (3) the Δ17-domain from PtDPL/PtRGA contains sites necessary for interaction. Although only half of the amino acid residues within the Δ17-domain of *PtDPL* and *PtRGA* are identical to those within the respective *A*. *thaliana* and rice genes, the GID-DELLA interaction does exist in the *P*. *tabuliformis* GA signalling pathway. Therefore, the GID1-mediated GA signalling cascade appeared after the divergence of vascular plants from the moss lineage^14^. The GA-GID1-DELLA signalling pathway has been gradually modified over the course of lycophyte, fern, conifer, and angiosperm evolution by changes within DELLA and GID proteins.

In conclusion, *PtGID1* acts as the GA receptor in *P*. *tabuliformis*. It is capable of interacting with DELLA proteins, and has the ability to recognise GA to form the GA-GID1-DELLA signalling module. This suggests that the GA signalling pathway operates in conifers, and is present in other vascular plants of substantially different evolutionary ages, such as in lycophytes, ferns, and angiosperms.

## Materials and Methods

### Plant material

*Pinus tabuliformis* tree cones were collected from genetically distinct trees selected at random in a primary clonal seed orchard located in Xingcheng City, Liaoning Province, China^30^. Details about this seed orchard can be found in a previous study^40^. Seeds from wild-type *A*. *thaliana* Col, and from transformants in which the *PtGID1* gene was overexpressed, were used in this study. All genotypes were in the Columbia background. Prior to germination, seeds were washed in 84% hydrogen peroxide and alcohol, then washed again five times with sterile water. All seeds were germinated on Murashige and Skoog (MS)-agar supplemented with 20 μM PAC, 40 μM PAC, or 40 μM PAC and 1 μM GA_3_, and incubated for 15 or 25 d at 22 °C. For root growth experiments, all seeds were grown on MS-agar supplemented with 0, 0.2, 0.5, or 1 μM GA_3_ and stacked vertically in a growth chamber (22 °C; 16-h photoperiod). Root length was measured from root tip to the base of the hypocotyl. For salt tolerance experiments, seeds were thoroughly washed as described above and grown on MS-agar medium for 6 d. Subsequently, seedlings were transferred to MS-agar supplemented with 150 mM NaCl and incubated at 22 °C for 20 d. For gene expression analysis, pine seedlings were irrigated with 50 μM GA_3_, 50 μM GA_4+7_, or 50 μM PAC for 2 d.

### Identification, cloning, and *in silico* protein structural analysis of *GID1* and *DELLA* genes from *P*. *tabuliformis*

The protein-encoding gene sequences of *GID1* in *A*. *thaliana*, *AtGID1a* (AT3G05120), *AtGID1b* (AT3G63010), and *AtGID1c* (AT5G27320), and in rice, *OsGID1* (Os05g0407500), were used to screen the *P*. *tabuliformis* reference transcriptome^30^ for homologous EST sequences. Full-length coding sequences from *P*. *tabuliformis* were cloned based on high homology. Next, we performed a reverse database search at The Arabidopsis Information Resource (TAIR) by contrasting these GID1-homologous sequences from *P*. *tabuliformis* with those from the *A*. *thaliana* genome, using TBLASTN to obtain homologous sequences in *A*. *thaliana*. Subsequently, we carried out multiple alignment of full-length protein sequences using MUSCLE^41,42^. We used these protein sequences to build a maximum-likelihood (ML) phylogenetic tree based on the JTT model to identify the true orthologues of GID1. SWISS-MODEL (http://swissmodel.expasy.org) was used to generate 3D protein models^43^ on the basis of the known X-ray crystal structure profile for rice GID1.

The protein-coding gene sequences of DELLAs in *A*. *thaliana*, *AtRGA* (AT2G01570), *AtGAI* (AT1G14920), *AtRGL2* (AT3G03450), *AtRGL3* (AT5G17490), and *AtRGL1* (AT1G66350), were used to screen the *P*. *tabuliformis* reference transcriptome^30^ for homologous EST sequences. The *P*. *tabuliformis* full-length mRNA sequences of hits were obtained, and were further translated *in silico*. We constructed an ML phylogenetic tree, including the *P*. *tabuliformis* sequences as well as full-length protein sequences from *rice*, *Physcomitrella patens*, and *S*. *moellendorffii* (OsSLR1 [Os03g49990], PpGAL1 [XP_001754090], PpGAL2 [XP_001774314], and SmDELLA1 [XP_00296024]).

### Overexpression of the *PtGID1* gene in *A*. *thaliana*

Full-length cDNAs for *PtGID1* containing suitable restriction enzyme sites at both ends were prepared by PCR, then inserted into a pBI121 vector containing the constitutive 35S promoter. The *Spe*I site was used for cloning *PtGID1* and obtaining the p35S-PtGID1 fragment. The p35S-PtGID1 fragment was introduced into wild-type *A*. *thaliana* plants by *Agrobacterium*-mediated transformation^44^. Expression of the transgene in *A*. *thaliana* plants was confirmed by PCR. The primers used are listed in Table S1. Transgenic plants were grown in a greenhouse under a constant day length of 16 h.

### BiFC and infiltration in *A*. *thaliana* leaf tissue

For the bimolecular fluorescence complementation (BiFC) assay, *AtGID1* and *PtGID1* genes containing appropriate restriction sites at both ends were cloned into the pSPYNE vector, using the *Stu*I-*Xho*I sites for *AtGID1* and *PtGID1*, to produce pSPYNE-GID1 plasmids. Similarly, the entire coding regions of *PtDPL*, *PtRGA*, *AtGAI*, *Ptdpl*, *Ptrga*, and *Atgai* sequences were cloned into the pSPYCE vector, using the *Stu*I-*Xho*I site to produce pSPYCE-DELLA and pSPYCE-della plasmids. Table S1 lists the primers that were used. All expression vectors were introduced into *A*. *tumefaciens* LBA4404. Agrobacteria were incubated, harvested, and resuspended in agroinfiltration buffer (0.2 mM acetosyringone, 10 mM MgCl_2_, and 10 mM MES). Agroinfiltration buffer was mixed with an equal volume of the protein mixture and injected into *A*. *thaliana* leaves using a syringe. Seventy two hours after infiltration, images were taken using a Leica TCS SP5 confocal microscope.

### Gene expression analysis

Total RNA was extracted using the TRIzol reagent (Invitrogen, California, USA) from 8-d-old *Arabidopsi*s seedlings or 2-month-old *P*. *tabuliformis* pine needles. RNA yield was determined using a NanoDrop 2000 spectrophotometer (Thermo Scientific, USA), and its integrity was evaluated using agarose gel electrophoresis with ethidium bromide staining. Total RNA (0.5 μg) was reverse transcribed into cDNA in a GeneAmp PCR System 9700 (Applied Biosystems, USA). A 1-μl aliquot of cDNA was used in 10-μl reactions in the GeneAmp PCR System 9700 (Applied Biosystems, USA) using the LightCycler 480 II Real-time PCR Instrument (Roche, Swiss). Each sample was run in triplicate. At the end of the PCR cycles, melting curve analysis was performed to validate the generation of the expected PCR product. The gene-specific primers used are listed in Table S2. mRNA expression levels were normalised to 18S rRNA and were calculated using the 2^−ΔΔCt^ method^45^.

## Electronic supplementary material

supplementary information
