# Supplementary material for: The gibberellin GID1-DELLA signalling module exists in evolutionarily ancient conifers
Source: Sci Rep. 2017 Nov 30;7:16637. doi: 10.1038/s41598-017-11859-w (PMC5709395; doi:10.1038/s41598-017-11859-w)
Supplement: Supplementary file 1 — supplementary information [file 41598_2017_11859_MOESM1_ESM.pdf]

The gibberellin *GID1-DELLA* signaling module exists in the evolutionary ancient conifers

Ran Du[a], Shihui Niu[a], Ilga Porth[b], Yousry A El-Kassaby[c] and Wei Li[a,1]

Figure S1. Phylogenetic analysis of GID1-like genes from *P. tabuliformis* and *A. thaliana*.

The maximum likelihood tree is based on full-length protein sequences. The genes' names and IDs are provided on the right of each branch. Bootstrap values were obtained by running 1,000 bootstrap replicates. The horizontal branch lengths are proportional to the estimated number of aa substitutions per residue. The arrows indicate *P. tabuliformis* genes isolated in this study.

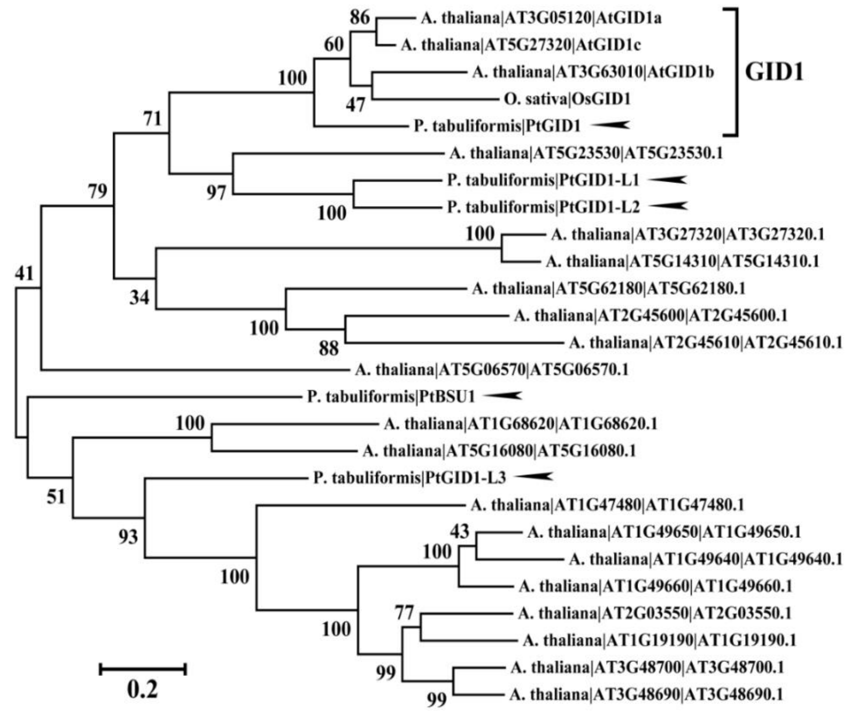

Figure S2. The structures of the rice *Os*GID1 and the respective homology modeling of *P. tabuliformis PtGID1* (E value: $2.17e^{-129}$, QMEAN Z-Score: -2.19).

The structures of the rice *Os*GID1 and the respective homology modeling of *P. tabuliformis PtGID1* (E value: $2.17e^{-129}$, QMEAN Z-Score: -2.19).

The red ball indicates the binding of gibberellic acid (GA). The N-terminal extension (N-Ex, blue alpha-helix) represents the GA binding-site of GID1. The structures on the right are their mirrored images.

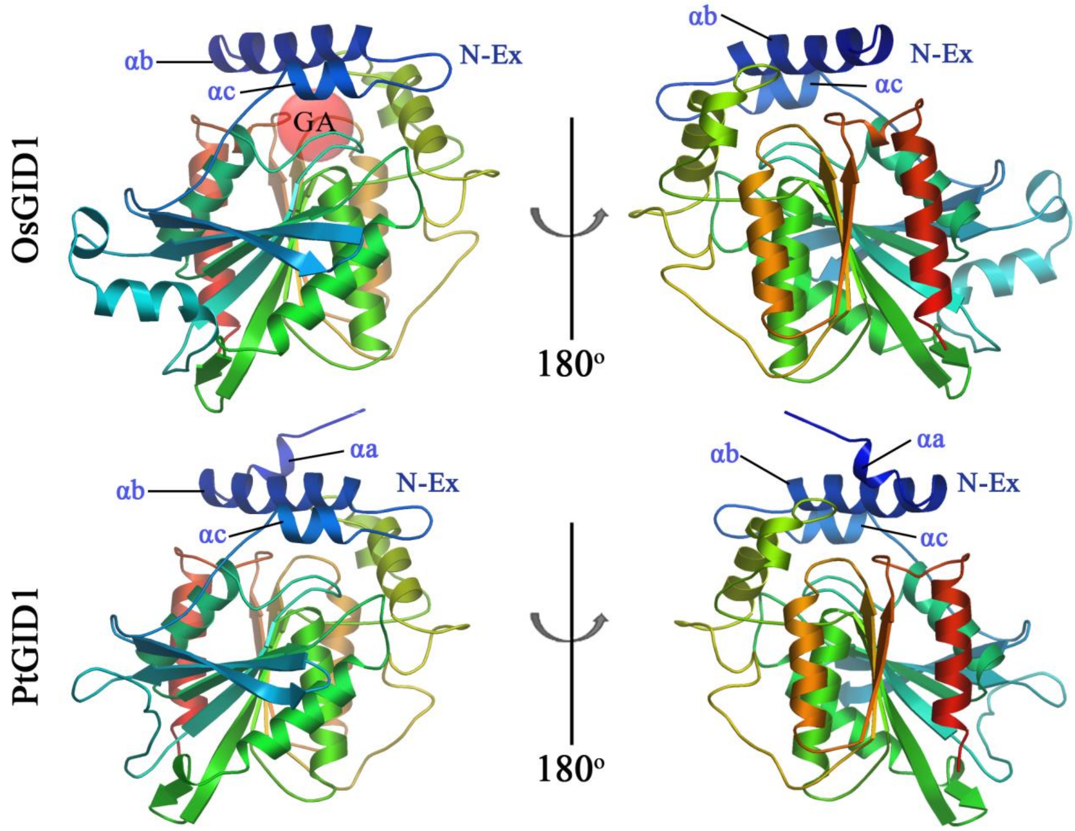

Table S1. Primers used in PCR reactions

Primer	Sequence
PtGID-F0	ATGGCTTCCAGCGATGATTATG
PtGID-Rs	CTCCGTCGACATTAACAGAATTCTCGGCGCCG
AtGID1a-Rs	CTCCGTCGACACATTCCGCGTTTACAAAC
AtGID1a-F0	ATGGCTGCGAGCGATGAAGTTA
PtDPL-F0	ATGAAGCGACAACACTTTCAAT
PtDPL-Rs	CTCCGTCGACACAGCGTTGCCAGGCAGAAGC
PtRGA-m-F0	ATGGACCCCATGGAAAGGGCTG
PtRGA-m-Rs	CTCCGTCGACGGAGCCTTGCCATGCAGATGCA
AtGAI-F	ATGAAGAGAGATCATCATCATC
AtGAI-Rs	CTCCGTCGACATTGGTGGAGAGTTTCCAAGCC

Table S2. GA biosynthesis gene primers

Primer	Sequence
18S-rRNA-F	AGTCATCAGCTCGCGTTGAC
18S-rRNA-R	TCAATCGGTAGGAGCGACG
GA2ox2-F	GGACCAAACGGTGACGTTG
GA2ox2-R	GTACTCCTCCACCGACTCACG
GA2ox4-F	GATGGCATGTGGGTTTCTGTC
GA2ox4-R	TCTCCCGTTCGTCATCACCT
GA20ox1-F	TTTCACCGGACGCTTCTCC
GA20ox1-R	CGCAAAACCGGAAAGAAAGG
GA20ox2-F	CGATCTCTCAAGCCAAGACTCG
GA20ox2-R	TCGCTGACGCCATGATTG
GA3ox1-F	TCCCGGATTCTTACAAGTGGAC
GA3ox1-R	GCCGGAGGAGAAGGAGCA
GA3ox2-F	CCCCTCCACGATTTCCGTA
GA3ox2-R	TGCGAACCACATCAACTTGG
PtGA3ox1-F	CAGAAGCAGAAGCAGACTTT
PtGA3ox1-R	ATAACAGTGGATGCTTGGAAT
PtGA3ox2-F	GCATCAAGCCCAGACTTT
PtGA3ox2-R	TATAAGAGAGGGTGCTTGGAAT
PtGA20ox1-F	AATAAGTGGCATTCTGTGCGA
PtGA20ox1-R	TGGAGGCAGCTCTTGTATTT
PtKAO1-F	TACAGGTGGTGGAATGCTC
PtKAO1-R	CTGTAGGTTTCGCCAATCAG
PtKAO2-F	AAATGCTGGGCATGAGTC
PtKAO2-R	CGATCTATCCTTCTCTTCACAA
PtGID1-F	CAACATCCTGCTGAACCC
PtGID1-R	CCAATCTCGGTCCCGTAT
